# Predictive Effect of Internet Addiction and Academic Values on Satisfaction With Academic Performance Among High School Students in Mainland China

**DOI:** 10.3389/fpsyg.2021.797906

**Published:** 2021-12-15

**Authors:** Diya Dou, Daniel T. L. Shek

**Affiliations:** Department of Applied Social Sciences, The Hong Kong Polytechnic University, Hong Kong, Hong Kong SAR, China

**Keywords:** internet addiction, academic value, satisfaction with academic performance, high school students, mainland China

## Abstract

Academic performance occupies an important role in adolescent development. It reflects adolescents’ cognitive ability and also shapes their academic and career paths. Students who are satisfied with their school performance tend to show higher self-esteem, confidence, and motivation. Previous research has suggested that students’ problem behaviors, such as Internet Addiction (IA), and academic values, including intrinsic and utility values, could predict satisfaction with academic performance. However, the influence of IA and academic values has not been thoroughly explored in Chinese contexts where the pressure for academic success is heavy. This study examined the relationships between IA, academic values (intrinsic and utility value), and satisfaction with academic performance using two waves of data collected from secondary school students in four cities in mainland China. The matched sample included a total of 2,648 Grade 7 or 8 students (57.1% were boys with a mean age of 13.1 years at Wave 1). Participants completed the same questionnaire containing validated measures at both waves with a 1-year interval. In line with the hypotheses, multiple regression analyses showed that Wave 1 IA was a significant negative predictor of Wave 2 intrinsic value, utility value, and satisfaction with academic performance and their changes. Results of mediation analyses revealed that only intrinsic value, but not utility value, positively predicted satisfaction with academic performance. Structural equation modeling (SEM) analyses also showed similar findings. Two observations are concluded from the present findings: IA impaired students’ intrinsic value, utility value, and perceived satisfaction with academic performance; two aspects of academic values demonstrated different influences on satisfaction with academic performance. These findings provide implications for the promotion of academic satisfaction experienced by students and the prevention of negative effects of IA.

## Introduction

The Internet has significantly changed people’s lives nowadays. Despite the profound benefits of the Internet, the public is aware of the negative influence of its overuse of misuse on health and well-being. One common problem is Internet addiction (IA), which refers to one’s inability to control Internet use that consequently causes social, psychological, academic, and work difficulties in life (Chou and Hsiao, 2000). IA has drawn growing concerns of the public and professionals worldwide.

Among different age groups, adolescents are considered more vulnerable to IA as their cognitive ability, self-control, and coping strategies are not fully developed (Long et al., 2018). Many studies have revealed that adolescents have a higher tendency to develop addictive behaviors such as playing online games or using social media in comparison to adults (Long et al., 2018). As the Internet penetration rate has dramatically increased nowadays, more than 80% of the adolescent population in the United Kingdom, United States, and Asia can access the Internet (Cerniglia et al., 2017). According to a national report, around 940 million Chinese people were Internet users, and among them were 172 million children and adolescents (China Internet Network Information Center, 2020). Research has revealed a relatively high prevalence of IA among Chinese adolescents. Shek et al. (2008) conducted research with 6,121 Chinese primary and high school students in Hong Kong, revealing that around 20% of the respondents met the criteria for IA based on two assessment measures. The study of Tan et al. (2016) involving 1,772 high school students in southern China also showed that around 17.2% of participants demonstrating problematic Internet use.

Many studies have documented the negative impact of IA on different aspects of adolescent development, such as sleeping quality (Tan et al., 2016), mental health (Ko et al., 2012), subjective well-being (Allen and Anderson, 2018), social development (Cerniglia et al., 2017), emotional development (Truzoli et al., 2020), and interpersonal relationship (Zeng et al., 2021). For adolescents, IA is particularly associated with low levels of school performance. Empirical evidence showed that students with IA experience more academic failure than their counterparts (Nemati and Matlabi, 2017). For example, students’ online gambling habits were positively related to low levels of school achievements and less prosocial behaviors (Floros et al., 2015). Online pornography watching also impaired adolescents’ academic performance as it reduces their interest, concentration, and involvement in academic activities (Beyens et al., 2015). Similar results were found among Chinese adolescents. For example, a longitudinal study examined the relationship between Internet behavior and students’ academic development based on a sample of 9,949 Chinese students revealed that IA could lead to lower academic achievement, dropout, and absenteeism (Anthony et al., 2021). Another study evaluating IA and negative emotions also reported that IA negatively influenced academic problems by undermining students’ mental and psychological health (Bu et al., 2021). The study of Bai et al. (2020) based on 1,794 adolescents from low-income families in China revealed that IA was linked to depression and detrimental to students’ academic performance.

In Chinese schools, students are evaluated publicly by peers and teachers in terms of whether their behavioral and academic performance reaches school standards, which largely influences students’ psychological health and adjustment (Chen et al., 2012). Undoubtedly, academic performance is considered the most important standard in Chinese school context. Researchers have adopted different approaches to assess academic performance. Primarily, test scores are considered an objective indicator of academic performance and have been often used in previous studies. Although the use of test scores is helpful to suggest education improvement and school accountability, researchers have questioned whether test scores reflect the stable status of individual students’ overall development (Goldhaber and Özek, 2019). An alternative is to use subjective indicators, such as perceived performance level, which reflects one’s overall subjective evaluation of normative performance level compared to peers (Saw et al., 2016). Researchers have pointed out the importance of subjective perceptions of one’s academic performance for its close association with students’ psychological adjustment (Haraldsen et al., 2020). Researchers also argued that satisfaction with perceived academic performance as an element of school adjustment provides a better indication of one’s appraisal of academic achievement in schools (Shek, 2002). Research has shown that dissatisfaction with one’s academic performance constitutes developmental problems for adolescents, particularly when the failure occurs repetitively (Enns et al., 2001; Lee et al., 2016). As the present study was interested in the roles of perceived academic values and motivation, we used satisfaction with academic performance as the indicator.

Scientific studies have been conducted to unravel the mechanisms of the negative impacts of IA on academic performance among adolescents. Earlier research has focused on the distraction and divergence behaviors in learning among students with IA, which often directly lead to a decline in school performance. Besides, anxiety and depression have been found to mediate the adverse effect of IA on academic performance (Ko et al., 2012; Bai et al., 2020; Bu et al., 2021). Recent evidence suggests that IA may also interrupt students’ psychological learning process and create problems in academic values and motivation (Reed and Reay, 2015; Truzoli et al., 2020). For example, problematic Internet use was found to exert a negative effect on academic motivation, learning productivity, and psychosocial status, which have negative effects on academic performance (Truzoli et al., 2020).

Academic motivation includes intrinsic value and utility value (Eccles, 1983; Neel and Fuligni, 2013). Intrinsic value involves a sense of satisfaction rooted in the study or learning procedure itself, while utility value refers to students’ sense of the instrumental value of the school courses (such as getting higher grades or material rewards) rather than finding the courses interesting. Ryan and Deci (2000) also categorized motivation into intrinsic motivation and extrinsic motivation. Intrinsic motivation refers to an individuals’ aspiration for doing something from the inner heart, while extrinsic motivation defines the concept of getting rewards from outside to stimulate someone to behave (Benabou and Tirole, 2003).

Previous studies have found that IA may impair intrinsic value, as studying is often not as attractive as surfing the Internet (Hanus and Fox, 2015; Reed and Reay, 2015). The various attractive and interesting sensory stimulations derived from the Internet could undermine students’ learning interest, self-control, and self-efficacy in learning. Wang et al. (2021) argued that problematic use of short-form video applications was associated with a sole focus on immediate hedonic rewards and a lack of understanding of future harmful consequences. The research of Anthony et al. (2021) found a close relationship between IA and a lack of interest in school learning. A study conducted with Chinese students also revealed the mediating role of intrinsic motivation in the positive relationship between social media use and academic performance (Malik et al., 2020). Previous studies have mainly focused on the negative influence of IA and intrinsic value but paid less attention to the relationships between IA and utility value. Theoretically speaking, IA could also undermine utility value or extrinsic motivation as the intensive reinforcement and reward schedules in Internet activities (e.g., online games) provide instant extrinsic rewards to adolescents (Truzoli et al., 2020), while students may not necessarily receive instant extrinsic rewards (e.g., high grades or praise) even if they study hard.

Although IA has been commonly considered a risk factor for academic values and performance, how the two types of academic values are associated with performance are less conclusive. Theoretically speaking, intrinsic value promotes academic performance through positive and active engagement in learning with enjoyment, autonomy, deep learning, task arrangement, and time spending in learning (Vansteenkiste et al., 2005; Froiland and Oros, 2014; Liu et al., 2020). Intrinsic value is considered to have a relatively long-term effect on academic performance because it reinforces students’ self-concepts and values, which are vital for students to maintain healthy psychological status and deal with academic failures (Cheo, 2017). On the contrary, utility value is constrained by the existence of external rewards and thus believed to have an instant but short-term positive effect on academic performance. In other words, once external rewards are terminated, utility value may become ineffective in stimulating adolescents’ continuous efforts into their study.

However, empirical evidence supporting the distinctive effects of the two types of values has been equivocal. For example, the study of Baker (2004) on university students found no significant relationships between intrinsic or extrinsic motivation and academic achievement. Some studies revealed that both intrinsic and utility value were positively linked to school performance (Afzal et al., 2010). Some other studies revealed differential effects of intrinsic and utility value on academic outcomes (Moneta and Siu, 2002). For example, a longitudinal study conducted with 13,799 Chinese high school students revealed the different effects of intrinsic and utility value on academic performance. Students with high levels of intrinsic value were more attentive, focused on learning interests, arranged flexible learning strategies, and spent more time learning to improve their academic performance (Liu et al., 2020). It was argued that utility value might undermine the academic performance of students with high intrinsic value because utility value made students feel of being controlled, which damaged ones’ intrinsic values (Wang and Guthrie, 2004; Liu et al., 2020). Similarly, Kuvaas et al. (2017) found that intrinsic value was positively associated with better performance, while extrinsic motivation showed a modest negative effect on performance. These inconclusive findings call for further exploration on how academic values might be differently related to adolescent development.

This study aimed to fill some research gaps. First, this study explored the effects of IA on perceived satisfaction of academic performance and the mediating roles of both intrinsic and utility values. This helps reveal the underlying mechanism of the influence of IA on academic performance and clarify the function of two types of academic values, which would fill the above-mentioned theoretical gaps. Second, most existing studies on adolescent IA and academic outcomes have been conducted with Western samples, hence calling for devoting more efforts to these issues in non-Western societies, particularly in Chinese contexts (Shek et al., 2008; Shek and Yu, 2016). As academic excellence is highly emphasized in Chinese societies, academic motivation may be perceived differently. In fact, both intrinsic and utility values are emphasized in traditional Chinese culture. Regarding intrinsic value, Confucian stated that “wasn’t it a pleasure to learn and practice often?” (“xue er shi xi zhi, bu. yi yue hu?”) in the Analects of Confucius, highlighting the satisfaction of learning, practical application, and self-improvement (Waley, 2005). As to utility value, the Chinese saying, “one who excels in the study can follow an official career” (“xue er you ze shi”), emphasizes the benefits of academic excellence in future career development. In contemporary Chines societies, “an official career” may no longer be the ultimate goal of studying. However, the value of education still receives great recognition among the public despite the development of ideology and philosophy in China (Wang and Ross, 2010). At the national level, China’s Education Modernization 2035 plan sets the direction for developing the education sector to strengthen its overall capacity and international influence and makes China a powerhouse of education, human resources, and talents. At the family and individual levels, parents and students believe that “knowledge changes fate” and thus highly emphasize academic success (Xiang, 2018). Third, as most studies have not collected longitudinal data, it is difficult to establish the causal relationships between IA and academic performance. In particular, although longitudinal studies have examined the antecedents of IA (e.g., Yu and Shek, 2013), limited research has examined the longitudinal prediction of IA on adolescent developmental outcomes in Chinese adolescents. This research aims to understand the relationship between IA and academic performance and examine the mediating role of academic motivation (intrinsic and utility values) in this relationship using two waves of data.

## Research Hypotheses

Based on the literature, we proposed the following hypotheses for each research question.

### Research Question 1 (RQ1)

What are the concurrent and longitudinal relationships between IA and academic motivation? Based on the previous findings (Truzoli et al., 2020), we proposed that IA would be negatively associated with intrinsic value concurrently (Hypothesis 1a) and longitudinally (Hypothesis 1b). Besides, with reference to the existing literature (Ryan and Deci, 2000; Truzoli et al., 2020), we expected negative concurrent and longitudinal relationships between IA and utility value (Hypotheses 1c,d, respectively).

### Research Question 2 (RQ2)

What are the concurrent and longitudinal relationships between IA and satisfaction with academic performance? In line with studies conducted with Chinese students (Anthony et al., 2021), we proposed that IA would be negatively related to satisfaction with academic performance concurrently (Hypothesis 2a) and longitudinally (Hypothesis 2b).

### Research Question 3 (RQ3)

What are the concurrent and longitudinal relationships between academic motivation and satisfaction with academic performance? In line with previous research (Anthony et al., 2021), we proposed that intrinsic value would be positively linked to satisfaction with academic performance concurrently (Hypothesis 3a) and longitudinally (Hypothesis 3b). Similarly, utility value would also show positive associations with satisfaction with academic performance concurrently (Hypothesis 3c) and over time (Hypothesis 3d).

### Research Question 4 (RQ4)

Does academic motivation mediate the relationship between IA and satisfaction with academic performance? According to previous studies suggesting the mediating role of academic motivation (Malik et al., 2020), we hypothesized that intrinsic value and utility value would mediate the impact of IA on satisfaction with academic performance (Hypotheses 4a,b, respectively).

## Materials and Methods

### Participants and Procedure

The data of this study were derived from a project examining adolescent adjustment and development in mainland China. The participants were recruited from four junior high schools in three provinces. Two waves of data were collected at the beginning of the school year of 2016/2017 (Wave 1) and 1 year later (Wave 2). A survey questionnaire was administered to students during school hours. Students were informed of the research aims, data collection, and the principles that the data collected will be anonymous, confidential, and only used for academic purposes. We obtained written consent from students, their parents, teachers, and school heads before data collection. This study has been reviewed and granted ethical approval by the authors’ university.

In total, 3,010 students completed the questionnaire at Wave 1. Among them, 1,362 were in Grade 7, and 1,648 were in Grade 8. The data at Wave 2 were collected from 2,648 students, including 1,305 Grade 8 students and 1,343 Grade 9 students. The matched sample consisted of 2,648 students (Boys = 1,513; Girls = 1,109) with a mean age of 13.12 years at Wave 1. The attrition rates were 4.2 and 18.5% for Grade 7 and Grade 8 students, respectively, which were more favorable compared with studies reported in longitudinal studies with adolescents (Epstein and Botvin, 2000). Results of attrition analysis revealed non-significant differences between students in the matched sample (*n* = 2,648) and the dropouts (*N* = 362) in terms of age, IA, intrinsic and utility values, and satisfaction with academic performance in both grades.

### Measures

#### Internet Addiction

The Chinese version of the Internet Addiction Scale developed by Young (1998) was adopted to evaluate participants’ IA symptoms. This scale has been used and validated in previous studies and showed good psychometric properties (Shek et al., 2008; Yu and Shek, 2013; Chi et al., 2020). It includes 10 items assessing different IA symptoms, such as “Have you lied to family members, teachers, social workers, or others to conceal the extent of involvement with the Internet?” Participants indicated whether they exhibited each of the symptoms in the past 12 months on a dichotomous scale (i.e., yes/no). The total score equals the counts of “yes” answers to 10 questions. The values of Cronbach’s *α* of IA were 0.77 at Wave 1 and 0.80 at Wave 2.

#### Academic Values

Students’ academic values were measured *via* two aspects, including intrinsic value and utility value (Eccles, 1983; Neel and Fuligni, 2013). This scale has been validated in the Chinese context (Guo et al., 2017). Intrinsic value depicts how students perceive schoolwork as interesting and how much they like schoolwork in general. It includes two items: “In general, I find working on schoolwork is…” (1 = “very boring” and 5 = “very interesting”) and “How much do you like working on schoolwork?” (1 = “a little” and 5 = “a lot”). On the other hand, the utility value describes the perceived usefulness of schoolwork through three items: “Right now, how useful do you find things you learn in school to be in your everyday life,” “In the future, how useful do you think the things you have learned in school will be in your everyday life?” and “How useful do you think the things you have learned in school will be for what you want to be after you graduate” on a five-point Likert scale (1 = “not useful at all” and 5 = “very useful”). The Cronbach’s *α* estimates for the two scales ranged between 0.87 and 0.91 at the two waves, suggesting good internal consistency of the scales in this study.

#### Satisfaction With Academic Performance

Satisfaction with academic performance was measured by a single item, “I am satisfied with my academic performance as compared to my classmates,” on a six-point reporting scale (“1 = strongly disagree”; “6 = strongly agree”). This item was developed by authors based on literature (Education and Manpower Bureau, 2003) and has been used in previous studies (Shek, 2002).

### Data Analysis

We first conducted descriptive analyses. Table 1 summarizes the means, SDs, and correlations among variables. Hierarchical multiple regression analyses were conducted to examine the concurrent and longitudinal relationships between research variables (RQ1, RQ2, and RQ3). This approach has been commonly adopted in the field (Zhou et al., 2020; Dou and Shek, 2021). Particularly, we examined the longitudinal effects of IA at Wave 1 on academic outcomes at Wave 2 with the corresponding outcomes at Wave 1 controlled. By controlling the influence of the initial levels of academic outcomes, this method suggests the effect of the predictor variables on the dependent variables over time.

**Table 1:** Descriptive and correlational analyses.

Measures	Mean	*SD*	Correlations									
			1	2	3	4	5	6	7	8	9	10
Age	13.124	0.809										
Gendera	−	−	−0.081^***^									
Family intactnessb	−	−	0.019	0.009								
W1 IA	2.308	2.344	0.088^***^	−0.168^***^	0.081^***^							
W2 IA	2.300	2.434	0.057^**^	−0.097^***^	0.051^**^	0.451^***^						
W1 Intrinsic value	3.386	1.356	−0.094^***^	0.012	−0.066^***^	−0.304^***^	−0.217^***^					
W2 Intrinsic value	3.292	1.304	−0.050^**^	−0.010	−0.042^*^	−0.200^***^	−0.269^***^	0.411^***^				
W1 Utility value	4.145	0.988	−0.097^***^	0.039^*^	−0.080^***^	−0.288^***^	−0.184^***^	0.514^***^	0.275^***^			
W2 Utility value	3.929	1.036	−0.073^***^	0.008	−0.052^**^	−0.181^***^	−0.215^***^	0.337^***^	0.625^***^	0.388^***^		
W1 Academic performance satisfaction	3.781	1.483	−0.092^***^	−0.101^***^	−0.042^*^	−0.139^***^	−0.088^***^	0.226^***^	0.131^***^	0.154^***^	0.127^***^	
W2 Academic performance satisfaction	3.967	1.484	−0.066^***^	−0.126^***^	−0.028	−0.087^***^	−0.172^***^	0.199^***^	0.232^***^	0.138^***^	0.166^***^	0.269^***^

a 1 = boy, 2 = girl.

b 1 = intact, 2 = non-intact; W1 = Wave 1; W2 = Wave 2; and IA = Internet addiction.

* *p* < 0.05;

** *p* < 0.01;

*** *p* < 0.001.

For RQ4, we first analyzed the mediational role of intrinsic and utility value through a series of regression models using PROCESS macro in SPSS (Hayes, 2017). We calculated bias-corrected (BC) bootstrap 95% CIs using 2,000 re-samplings in the mediation analyses (Hayes, 2017). We first examined the mediating effects of intrinsic and utility values in two models separately, and then simultaneously added them to one model. This conservative method is helpful to explore the relationships between research variables in line with research questions and also suggest potential interactions. Besides, we used Structural Equation Modeling (SEM) to test the complete hypothesized model *via* Lavaan package in R software (Rosseel, 2012). SEM models can accommodate latent variables, multiple predictors, and outcomes, which allow a comprehensive analysis of the relationships between research variables. Multiple indices were used to indicate model goodness of fit, including Comparative Fit Index (“CFI”), Tucker-Lewis Index (“TLI”), Root Mean Square Error of Approximation (“RMSEA”), and Standardized Root Mean Square Residual (“SRMR”). Based on Hu and Bentler (1999) and Kline (2015), the cutoff criteria should be above 0.90 for CFI and TFI values, and lower than 0.08 for RMSEA and SRMR values.

## Results

### Descriptive Results and Correlations

Table 1 shows the means, SDs, and correlations for IA, intrinsic value, utility value, and satisfaction with school performance over the two time points. The correlations between the research variables were significant and in line with the hypotheses. IA was negatively associated with intrinsic and utility value concurrently and longitudinally (*r* ranged between −0.20 and −0.30, *p*s < 0.001), and was negatively correlated with satisfaction with academic performance at each wave (*r*s ranged between −0.09 and −0.17, *p*s < 0.001). Both intrinsic and utility values were positively correlated with satisfaction with academic performance at two waves (*r*s ranged between 0.127 and 0.232, *p*s < 0.001).

### Predictive Effects of IA on Academic Values

Results of hierarchical multiple regression analyses revealed significant concurrent negative effects of IA on intrinsic value (Wave 1: *b* = −0.30, *p* < 0.001, Cohen’s *f ^2^* = 0.096; Wave 2: *b* = −0.27, *p* < 0.001, Cohen’s *f*^2^ = 0.080, see Table 2) and utility value (Wave 1: *b* = −0.28, *p* < 0.001, and Cohen’s *f*^2^ = 0.079; Wave 2: *b* = −0.21, *p* < 0.001, and Cohen’s *f*^2^ = 0.046, see Table 3) at each wave after controlling gender, age, and family intactness. As to the longitudinal effect, Wave 1 IA had significant longitudinal effects on Wave 2 intrinsic value (*b* = −0.21, *p* < 0.001, and Cohen’s *f*^2^ = 0.042, see Table 4) and Wave 2 utility value (*b* = −0.28, *p* < 0.001, and Cohen’s *f*^2^ = 0.079, see Table 5). Additionally, after controlling Wave 1 intrinsic and utility values, IA at Wave 1 significantly predicted a decrease in both academic values over time (*b* was −0.09 and −0.21, *p*s < 0.001, and Cohen’s *f*^2^ was 0.007 and 0.046 for intrinsic and utility value, respectively, see Tables 4, 5). Hypotheses 1a, 1b, 1c, and 1d were supported.

**Table 2:** Cross-sectional regression analyses for intrinsic value.

Model	Predictors	Intrinsic value (Wave 1)					Intrinsic value (Wave 2)				
		*β*	*t*	Cohen’s *f^2^*	*R*^2^ change	*F* change	*β*	*t*	Cohen’s *f^2^*	*R*^2^ change	*F* change
1	Age	−0.09	−4.77^***^	0.009	0.012	10.52^***^	−0.05	−2.42^*^	0.002	0.004	3.77^*^
	Gender^a^	0.00	0.21	0.000			−0.01	−0.68	0.000		
	Family intactness^b^	−0.05	−2.78^**^	0.003			−0.04	−2.22^*^	0.002		
2	IA	−0.30	−15.98^***^	0.096	0.088	255.35^***^	−0.27	−14.52^***^	0.080	0.074	210.75^***^

In model 2, control variables were statistically controlled. IA at Wave 1 and Wave 2 were included as predictors to predict academic performance at Wave 1 and Wave 2, respectively.

a 1 = boy, 2 = girl.

b 1 = intact, 2 = non-intact. IA = Internet Addiction.

* *p* < 0.05;

** *p* < 0.01;

*** *p* < 0.001.

**Table 3:** Cross-sectional regression analyses for utility value.

Model	Predictors	Utility value (Wave 1)					Utility value (Wave 2)				
		*β*	*t*	Cohen’s *f^2^*	*R*^2^ change	*F* change	*β*	*t*	Cohen’s *f^2^*	*R*^2^ change	*F* change
1	Age	−0.09	−4.65^***^	0.008	0.016	14.57^***^	−0.07	−3.43^***^	0.005	0.007	6.14^***^
	Gender^a^	0.03	1.66	0.001			0.00	0.11	0.000		
	Family intactness^b^	−0.08	−4.1^***^	0.006			−0.05	−2.44^*^	0.002		
2	IA	−0.28	−14.54^***^	0.079	0.074	211.49^***^	−0.21	−11.07^***^	0.046	0.044	122.46^***^

In model 2, control variables were statistically controlled. IA at Wave 1 and Wave 2 were included as predictors to predict academic performance at Wave 1 and Wave 2, respectively.

a 1 = boy, 2 = girl.

b 1 = intact, 2 = non-intact. IA = Internet Addiction.

* *p* < 0.05;

*** *p* < 0.001.

**Table 4:** Longitudinal regression analyses for intrinsic value.

Model	Predictors	Intrinsic value (Wave 2)					Intrinsic value (Wave 2)				
		*β*	*t*	Cohen’s *f^2^*	*R*^2^ change	*F* change	*β*	*t*	Cohen’s *f^2^*	*R*^2^ change	*F* change
1	Age	−0.047	−2.42^*^	0.002	0.004	3.77^*^	−0.009	−0.48	0.000	0.170	540.66^***^
	Gender^a^	−0.013	−0.68	0.000			−0.015	−0.84	0.000		
	Family intactness^b^	−0.043	−2.22^*^	0.002			−0.021	−1.17	0.000		
	W1 intrinsic value						0.415	23.25^***^	0.206		·
2	W1 IA	−0.21	−10.51^***^	0.042	0.040	110.54^***^	−0.09	−4.61^***^	0.007	0.007	21.22^***^

In model 2, control variables were statistically controlled.

a 1 = boy, 2 = girl.

b 1 = intact, 2 = non-intact. W1 = Wave 1. IA = Internet addiction.

* *p* < 0.05;

*** *p* < 0.001.

**Table 5:** Longitudinal regression analyses for utility value.

Model	Predictors	Utility value (Wave 2)					Utility value (Wave 2)				
		*β*	*t*	Cohen’s *f^2^*	*R*^2^ change	*F* change	*β*	*t*	Cohen’s *f^2^*	*R*^2^ change	*F* change
1	Age	−0.067	−3.43^***^	0.004	0.007	6.14^***^	−0.032	−1.77	0.001	0.148	458.77^***^
	Gender^a^	0.002	0.11	0.000			−0.010	−0.57	0.000		
	Family intactness^b^	−0.048	−2.44*	0.002			−0.017	−0.93	0.000		
	W1 utility value						0.388	21.42^***^	0.174		·
2	W1 IA	−0.17	−8.92^***^	0.030	0.029	79.5^***^	−0.07	−3.82^***^	0.005	0.005	14.6^***^

In model 2, control variables were statistically controlled.

a 1 = boy, 2 = girl.

b 1 = intact, 2 = non-intact. W1 = Wave 1. IA = Internet addiction.

* *p* < 0.05;

*** *p* < 0.001.

### Predictive Effects of IA on Satisfaction With Academic Performance

Results of multiple regression analyses demonstrated that IA had a significantly negative influence on satisfaction with academic performance at each wave (*b* was −0.15 and − 0.18, *p*s < 0.001, and Cohen’s *f*^2^ was 0.022 and 0.034 for Wave 1 and 2, respectively, see Table 6). In addition, IA showed significant and negative prediction on Wave 2 satisfaction with academic performance (*b* = −0.11, *p* < 0.001, and Cohen’s *f*^2^ = 0.011, see Table 6). After controlling Wave 1 satisfaction with academic performance, IA significantly predicted a decrease in satisfaction with academic performance (*b* = −0.07, *p* < 0.001, and Cohen’s *f*^2^ = 0.004, see Table 6). Hypotheses 2a and 2b were supported.

**Table 6:** Cross-sectional regression analyses for academic performance.

Model	Predictors	Academic performance satisfaction (Wave 1)					Academic performance satisfaction (Wave 2)				
		*β*	*t*	Cohen’s *f^2^*	*R*^2^ change	F change	*β*	*t*	Cohen’s *f^2^*	*R*^2^ change	*F* change
1	Age	−0.10	−5.13^***^	0.010	0.022	18.93^***^	−0.08	−4.00^***^	0.006	0.023	20.08^***^
	Gender^a^	−0.11	−5.55^***^	0.012			−0.13	−6.79^***^	0.018		
	Family intactness^b^	−0.03	−1.73	0.001			−0.03	−1.35	0.001		
2	IA	−0.15	−7.64^***^	0.022	0.022	58.41^***^	−0.18	−9.48^***^	0.034	0.033	89.78^***^
	Intrinsic value	0.22	11.45^***^	0.050	0.048	131.04^***^	0.23	12.21^***^	0.057	0.054	149.11^***^
	Utility value	0.15	7.67^***^	0.022	0.022	58.87^***^	0.17	8.56^***^	0.028	0.027	73.2^***^

In model 2, control variables were statistically controlled. Predictors at Wave 1 and Wave 2 were included as predictors to predict academic performance at Wave 1 and Wave 2, respectively.

a 1 = boy, 2 = girl.

b 1 = intact, 2 = non-intact. IA = Internet Addiction.

*** *p* < 0.001.

### Predictive Effects of Academic Values on Satisfaction With Academic Performance

Results of multiple regression analyses revealed that intrinsic value and utility value positively predicted each wave’s satisfaction with academic performance (*b* ranged between 0.15 and 0.23, *p*s < 0.001, Cohen’s *f*^2^ ranged from 0.022 to 0.057, see Table 6). Results also showed a longitudinal prediction of intrinsic value and utility value on satisfaction with performance (*b* = 0.20 and 0.14, *p*s < 0.001, and Cohen’s *f*^2^ = 0.039 and 0.019 for intrinsic and utility value, respectively, see Table 7). Moreover, both intrinsic and utility values predicted an increase in Wave 2 satisfaction with academic performance when Wave 1 satisfaction was controlled (*b* = 0.15 and 0.10, *p*s < 0.001, and Cohen’s *f*^2^ = 0.020 and 0.010 for intrinsic and utility value, respectively, see Table 7). Results supported Hypotheses 3a, 3b, 3c, and 3d.

**Table 7:** Longitudinal regression analyses for academic performance.

Model	Predictors	Academic performance satisfaction (Wave 2)					Academic performance satisfaction (Wave 2)				
		*β*	*t*	Cohen’s *f^2^*	*R*^2^ change	*F* change	*β*	*t*	Cohen’s *f^2^*	*R*^2^ change	*F* change
1	Age	−0.076	−3.85^***^	0.006	0.023	20.12^***^	−0.050	−2.62^**^	0.002	0.063	173.07^***^
	Gender^a^	−0.135	−6.86^***^	0.019			−0.107	−5.55^***^	0.011		
	Family intactness^b^	−0.027	−1.35	0.001			−0.017	−0.91	0.000		
	W1 academic performance satisfaction						0.253	13.16^***^	0.067		·
2	IA	−0.11	−5.31^***^	0.011	0.011	28.24^***^	−0.07	−3.40^***^	0.004	0.004	11.54^***^
	Intrinsic value	0.20	10.14^***^	0.039	0.038	102.9^***^	0.15	7.53^***^	0.020	0.020	56.64^***^
	Utility value	0.14	7.07^***^	0.019	0.019	49.98^***^	0.10	5.37^***^	0.010	0.010	28.84^***^

In model 2, control variables were statistically controlled.

a 1 = boy, 2 = girl.

b 1 = intact, 2 = non-intact. IA = Internet addiction.

** *p* < 0.01;

*** *p* < 0.001.

### Mediating Roles of Academic Values

Results of mediation analyses *via* PROCESS are summarized in Table 8. When intrinsic and utility values were examined in two separate models, results revealed significant mediating effects of both intrinsic value (see Model 1a in Table 8) and utility value (see Model 1b in Table 8). When they were added to the model simultaneously, results showed that IA at Wave 1 negatively predicted intrinsic value and utility value at Wave 2. However, only intrinsic value, not utility value, positively predicted satisfaction with academic performance, suggesting the potential mediating effect of intrinsic value only (see Model 2 in Table 8). The indirect effect of IA on academic performance *via* intrinsic value was significant (*b* = −0.03, *p* < 0.001, see Model 2 in Table 8). The mediating effect of utility value was not significant (see Model 2 in Table 8).

**Table 8:** Longitudinal mediating effect analyses of intrinsic value and utility value at Wave 2 (the mediators) for the effect of IA at Wave 1 on academic performance satisfaction at Wave 2.

Regression models summary	Model 1a			Model 1b		
	Intrinsic value			Utility value		
	*β*	*SE*	*t*	*β*	*SE*	*t*
Total effect of IV on DV	−0.05	0.01	−4.43^***^	−0.05	0.01	−4.43^***^
IV to Mediator	−0.11	0.01	−10.48^***^	−0.08	0.01	−9.47^***^
Mediator to DV	0.25	0.02	11.46^***^	0.22	0.03	7.90^***^
Direct effect of IV on DV	−0.03	0.01	−2.16^*^	−0.04	0.01	−2.98^*^
Mediating effect	Point estimate	Bootstrapping (BC 95% CI)		Point estimate	Bootstrapping (BC 95% CI)	
		Lower	Upper		Lower	Upper	
	−0.04^***^	−0.06	−0.03	−0.00	−0.01	0.00
Regression models summary	Model 2 (intrinsic and utility values as mediators)					
	Intrinsic value			Utility value		
	*β*	*SE*	*t*	*β*	*SE*	*t*
Total effect of IV on DV	−0.09	0.01	−4.43^***^	−0.09	0.01	−4.43^***^
IV to Mediator	−0.20	0.01	−10.48^***^	−0.18	0.01	−9.47^***^
Mediator to DV	0.20	0.03	8.30^***^	0.03	0.03	1.27
Direct effect of IV on DV	−0.04	0.01	−2.07^*^	−0.04	0.01	−2.07^*^
Mediating effect	Point estimate	Bootstrapping (BC 95% CI)		Point estimate	Bootstrapping (BC 95% CI)	
		Lower	Upper		Lower	Upper	
	−0.03^***^	−0.03	−0.02	−0.00	−0.01	0.00

Model 1a and Model 1b examined the mediating effects of intrinsic and utility values separately. In Model 2, intrinsic value and utility value were simultaneously added to the model as mediators. BC, bias corrected; CI, confidence interval.

* *p* < 0.05;

*** *p* < 0.001.

**Figure 1:**
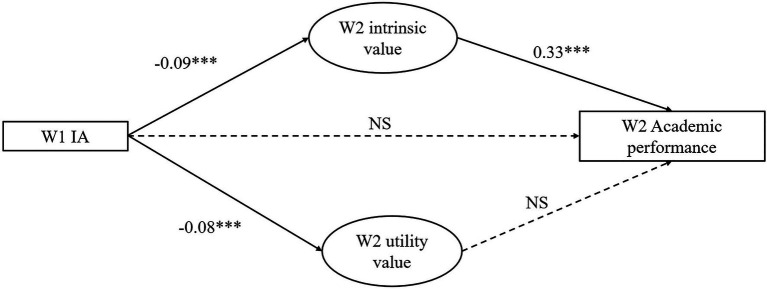
Results of Structural equation modeling (SEM) model. ***p < 0.001.

We further developed a SEM model to comprehensively understand the relationships between variables under investigation (see Figure 1). The SEM model included IA at Wave 1 and satisfaction with academic performance at Wave 2 as observed variables, and intrinsic and utility values at Wave 2 as latent variable. The SEM model showed adequate model fit (*χ*^2^ = 47.243, *df* = 9, CFI = 0.996, TFI = 0.990, NNFI = 0.990, RMSEA = 0.040, and SRMR = 0.011; Kline, 2015). Figure 1 shows the standardized coefficients in this model. IA at Wave 1 significantly and negatively predicted Wave 2 intrinsic value (*β* = −0.09, *p* < 0.001), utility value (*β* = −0.08, *p* < 0.001), but not satisfaction with academic performance (*p* = 0.064). Wave 2 intrinsic value, but not utility value, demonstrated a significant and positive prediction on academic performance (*β* = 0.33, *p* < 0.001). Results of SEM were in line with the PROCESS findings, which supported Hypothesis 4a but rejected Hypothesis 4b.

## Discussion

In this study, we examined the predictive effect of IA on satisfaction with academic performance, with academic values hypothesized as mediators. With reference to the research gaps in the literature, this study has several strengths. First, instead of focusing on objective academic performance indexed by test scores, we adopted students’ satisfaction with academic performance, an indication of students’ appraisal of overall academic achievement, to better understand the research questions concerning students’ psychological motivation and values. Second, this study examined two potential mechanisms through which having IA symptoms potentially predict students’ satisfaction with academic performance through intrinsic and/or utility value. Third, a short-term longitudinal design was used to understand the predictive effects of IA on satisfaction with academic performance. Fourth, we employed a relatively large sample to enhance the generalizability of the findings. Fifth, as very few studies in this field have been conducted in the Chinese context, this study contributes to the understanding of the negative influence of IA on academic performance and the underlying mechanisms in an educational system that highly emphasizes academic success. Finally, analyses based on both multiple regression and SEM were used to address research questions in a comprehensive manner.

Findings based on multiple regression analyses generally support the proposed hypotheses, which are consistent with the existing literature. First, IA negatively predicted satisfaction with academic performance and its change over time. The findings support previous evidence suggesting negative associations between IA and academic performance (Nemati and Matlabi, 2017; Anthony et al., 2021; Bu et al., 2021). Second, IA positively predicted both intrinsic and utility values and the changes over time. These findings are also in line with previous studies revealing negative influences of problem Internet use on students’ learning motivation (Truzoli et al., 2020; Anthony et al., 2021). Third, results of multiple regression showed that both intrinsic and utility values positively predicted satisfaction with academic performance and its change over time. Although some previous studies have emphasized the downside of utility value on adolescent development, the results of the present study corroborate previous evidence highlighting the positive influence of both intrinsic and utility values (Afzal et al., 2010). As mentioned earlier, Chinese cultures acknowledge both intrinsic and utility values of study. Although the education system in China has been criticized for the examination orientation, it is still perceived as the most accessible and fair approach for disadvantaged students to beat the odds and seek academic access (Wang and Ross, 2010). For these students, schooling means much more than individual interests or satisfaction but “a future of comfort and dignity, a family responsibility and collective investment, and a path toward individual freedom and actualization” (Xiang, 2018, p. 81). These beliefs reflect instrumental value but are also rooted in spirits of hard-working and persistence that are vital for academic success. Finally, when both intrinsic and utility values were included in the mediation models, only intrinsic value, but not utility value, served as a mediator in the relationship between IA and satisfaction with academic performance. Results based on multiple regression and SEM are consistent, which generate triangulated findings for the study. The results are consistent with the widely held belief that intrinsic and utility values are distinct constructs and have different associations with adolescents’ maladjustment and psychological well-being (Moneta and Siu, 2002). Students demonstrating more IA symptoms tended to regard school work as boring and consequently felt less satisfied with their academic performance, which is in agreement with previous findings (Liu et al., 2020). Additionally, the mediating effect of utility value was not significant when intrinsic value was taken into account. One explanation is that utility value may include different subtypes depending on how one internalizes the extrinsic goals as a personal pursuit. If students regard the striving for performance excellence as a personal commitment, it reflects high levels of autonomy and self-determination (Ryan and Deci, 2000). As results of the correlational analysis revealed a significant positive association between intrinsic and utility value, students may accept the utility of schooling and endorse the external goals. This finding echoes the idea that intrinsic value has an immediate effect on study performance, while ulitity value contributes to performance through its close association with intrinsic motivation (Wang and Guthrie, 2004). We should also investigate the linkages between the two types of academic value in future studies.

There are several theoretical implications of the present findings. First, the study suggests that the negative effects of IA on academic values and satisfaction with academic performance concurrently and over time, which strengthens the theoretical proposition that IA has longitudinal adverse effects on academic outcomes (Zhang et al., 2018). Second, the results underscore the importance of academic values, particularly intrinsic value, in mediating the influence of IA on satisfaction with academic performance. Students possessing high levels of intrinsic value perceive learning as exploratory, playful, and curiosity driven. According to Self-determination Theory (Ryan and Deci, 2000), intrinsic value serves as “a natural wellspring of learning.” However, many online activities, including short videos, social media networks, and online games, have been designed or presented to be mentally stimulating to give users high levels and continual enjoyment (King and Delfabbro, 2018). Students’ basic psychological needs for competence, autonomy, and relatedness may be better satisfied by Internet use rather than by traditional learning activities, which may lead to a decrease in their engagement in school work and an increase in Internet use (Salmela-Aro et al., 2017; Zhang et al., 2018). As existing research has paid much attention to the direct relationship between the Internet and academic performance, our results highlight the importance of examining how psychological factors mediate the relationship between adolescent problem behaviors and their development and well-being in the long run.

The finding has practical implications for teachers and social workers to help adolescents and their parents understand the negative consequences of IA in undermining academic values (i.e., meaning of study) and academic performance. Given that many teaching and learning activities are online nowadays, adolescents and parents commonly hold the belief that Internet is an indispensable part of life, and thus it cannot be addictive and the “prolonged” use of IA is not problematic. Instead, adolescents should be aware of the potential dark side of Internet use, such as the adverse effects of IA on academic values and perceived school performance (Salmela-Aro et al., 2017). Furthermore, to promote satisfaction with academic performance, we need to cultivate the meaning of studying in students. In school practices, it is trendy for teachers to adopt various pedagogical strategies to spark students’ intrinsic value and cultivate active learners. Utility value, on the contrary, is often regarded as ineffective or even detrimental in adolescent development and is often associated with unhealthy teaching or parenting styles, such as excessive involvement (Rivers et al., 2012). As Benabou and Tirole argued, “external incentives are weak reinforcers in the short run, and negative reinforcers in the long run” (2003, p. 489). However, our results did not reveal any negative associations between utility value and intrinsic value or academic performance. As suggested by Lin et al. (2003), we believe it is important that teachers and parents need not eliminate all perceived utility values for high performance, especially when students accepting utility value of schooling based on a sense of commitment and self-determination.

There are several limitations of the study. First, because only two waves of data were collected, the findings are based on a short-term longitudinal study. As such, more time points should be added in future studies. Second, the scale of academic values only included a few items for the two types of values. As suggested by Ryan and Deci (2000), it is meaningful to explore different subtypes of extrinsic motivation based on the perceived locus of causality. We recommend that more items and subtypes of utility value should be examined in future studies. Third, the present study only adopted a subjective indicator of academic performance. We believe satisfaction with performance better reflects adolescents’ self-evaluation on schooling and is closely associated with their psychological well-being. Although satisfaction with academic performance is closely correlated with GPA (Bradley, 2006), it would be helpful to include test scores and/or teacher-rated performance in future studies. Fourth, this study mainly focused on academic values as mediators. Other important factors, such as academic stress, could be taken into account in future studies (Baker, 2004). Finally, only self-report data were collected, which may lead to common-method variance bias. Future studies should use multiple informants’ reports to assess adolescent IA symptoms and academic performance.

## Data Availability Statement

The raw data supporting the conclusions of this article will be made available by the authors, without undue reservation.

## Ethics Statement

The studies involving human participants were reviewed and approved by Human Subjects Ethics Subcommittee at The Hong Kong Polytechnic University. Written informed consent to participate in this study was provided by the participants’ legal guardian/next of kin.

## Author Contributions

DS designed the research project and contributed to all the steps of the work. DD conducted data analyses, prepared the first draft, and revised the manuscript based on the comments and editing provided by DS. All authors contributed to the article and approved the submitted version.

## Funding

This paper and the two-wave longitudinal study in the Tin Ka Ping Project P.A.T.H.S. were financially supported by Tin Ka Ping Foundation. The APC was funded by a start-up grant to DD (Project ID: P0035101).

## Conflict of Interest

The authors declare that the research was conducted in the absence of any commercial or financial relationships that could be construed as a potential conflict of interest.

## Publisher’s Note

All claims expressed in this article are solely those of the authors and do not necessarily represent those of their affiliated organizations, or those of the publisher, the editors and the reviewers. Any product that may be evaluated in this article, or claim that may be made by its manufacturer, is not guaranteed or endorsed by the publisher.
